# Different extent in decline of infant mortality by region and cause in Shenyang, China

**DOI:** 10.1038/srep24527

**Published:** 2016-04-14

**Authors:** Yan-Hong Huang, Qi-Jun Wu, Li-Li Li, Da Li, Jing Li, Chen Zhou, Lang Wu, Jingjing Zhu, Ting-Ting Gong

**Affiliations:** 1 Department of science and education, Shenyang Women and Children Health Care Centre, Shenyang, China; 2 Department of Clinical Epidemiology, Shengjing Hospital of China Medical University, Shenyang, China; 3 Department of children’s health prevention, Shenyang Women and Children Health Care Centre, Shenyang, China; 4 Department of Obstetrics and Gynecology, Shengjing Hospital of China Medical University, Shenyang, China; 5 Department of information statistics, Shenyang Women and Children Health Care Centre, Shenyang, China; 6 Division of Epidemiology, Department of Medicine, Vanderbilt Epidemiology Center, Vanderbilt-Ingram Cancer Center, Vanderbilt University School of Medicine, Nashville, TN, USA

## Abstract

To compare the pattern of cause of death of infant mortality rates by urban/rural areas as well as to generate knowledge for potential strategies to decrease this mortality, we carried out a study by analyzing the infant mortality data from the Shenyang Women and Children Health Care Centre. From 1997 to 2014, 970,583 live births and 6510 infant deaths were registered. Infant mortality rates, percent change, and annual percent change (APC) were calculated. The infant mortality significantly decreased by 5.92%, 7.41%, and 3.92% per year in overall, urban, and rural areas, respectively. Among the categories of causes of infant death, congenital anomalies (APC = −7.87%), asphyxia-related conditions (APC = −9.43), immaturity-related conditions (APC = −3.44%), diseases of the nervous system and sense organs (APC = −6.01%), and diseases of the respiratory system (APC = −6.29%) decreased significantly in the observational periods. Additionally, among selective causes of infant death, pneumonia, congenital heart disease, neural tube defects, preterm birth and low birth weight, birth asphyxia, and intracranial hemorrhage of the newborn significantly decreased by 5.45%, 5.45%, 16.47%, 2.18%, 10.95%, and 10.33% per year, respectively. In conclusion, infant mortality has been continuously decreased in Shenyang from 1997 to 2014, although further efforts are still needed to decrease the infant mortality in rural areas.

The report “A Promise Renewed: 2015 Progress Report” from the United Nations Children’s Fund (UNICEF) noted that the number of children who die from mostly preventable causes before they turn five years old now stands at 5.9 million a year, decreased from 12.7 million 25 years ago^1^. Additionally, since 2000, the lives of 48 million children under the age of five have been saved after governments committed to achieving the Millennium Development Goals (MDGs), which are a set of development goals agreed upon by world leaders in 2000^1^. Despite the progress that has been made, one of the most prominent goals for 2015 (MDG-4), which aims to reduce the child mortality by two-thirds from the level in 1990, has not been satisfactorily met.

Infant mortality rate is an important indicator for the effectiveness of public health issues including maternal and child health care services, as well as for comparing countries with respect to welfare^2^. In the last two decades, China has made great progress in child health. For example, according to the report from UNICEF, the infant mortality in China declined from 42 per 1,000 live births in 1990 to 11 per 1,000 live births in 2013^3^. However, compared with developed countries (e.g. 2 and 5 per live births in Sweden and Canada in 2013, respectively), the infant mortality of China has not yet decreased to an acceptable level. Furthermore, besides effective medical technology, better access to pre- and postnatal care for all socioeconomic groups, and better nutrition^4^, factors contributing to the international variation in neonatal mortality rates largely stem from differences in the development of these countries. Similarly, in China, a huge gap exists in terms of people’s income and health status between urban and rural areas, and the gap may explain the regional differences in children’s health status and survival between urban and rural areas^5^. For example, although the average infant mortality rate was 15.3 per 1,000 live births in 2007 in China, the infant mortality rates in urban areas were much lower than those in rural areas (7.7 versus 18.6 per 1,000 live births). Therefore, the purpose of this study was to compare the pattern of cause of death of infant mortality rates by urban/rural areas as well as to generate knowledge for potential strategies to reduce infant deaths in the future on the basis of data collected through a relatively long observational period (1997–2014).

## Results

Table 1 presents the number of live births and infant deaths of the 18-year observational period. During this period, a total of 6,510 infants died (6.71 per 1,000 live births) in Shenyang. When stratified by areas, the infant mortality rates of urban areas were significantly lower than those of rural areas (5.10 versus 9.01 per 1,000 live births).

**Figure 1:**
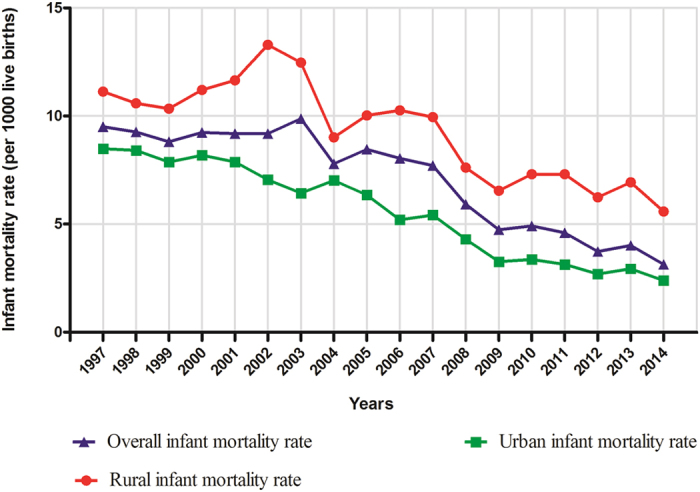
Trends in infant mortality rate (per 1000 live births) by areas in Shenyang, 1997–2014.

Figure 1 depicts the time trend and the overall infant mortality rates of Shenyang during the period of 1997–2014. The overall mortality of infants significantly decreased by 66.90% from 9.50 to 3.14 per 1,000 live births, or 5.92% per year (Table 2). Among the categories of causes of infant death, congenital anomalies (APC = −7.87%), asphyxia-related conditions (APC = −9.43), immaturity-related conditions (APC = −3.44), diseases of the nervous system and sense organs (APC = −6.01%), and diseases of the respiratory system (APC = −6.29%) decreased significantly in the observational period.

Stratifying by areas, we observed a significantly stronger decreasing trend of overall infant mortality in urban areas (APC = −7.41%) than in rural areas (APC = −3.92%) (Table 3). Compared with the findings in Table 2, although the same categories of causes of infant death were observed with significant decreasing trend in urban areas, as in the rural areas, the decreasing trend of diseases of the nervous system and sense organs did not show statistical significance (APC = −2.18%). Table 4 presents the contribution rates of each category of causes of death.

Table 4 shows the results of additional analyses for the selected causes of infant death. We observed significantly decreasing trends of all these selective causes. When stratified by areas, besides accidental asphyxia, the decreasing trends of preterm birth and low birth weight in both urban and rural areas as well as congenital heart disease in rural areas did not show statistical significances (Table 5).

## Discussion

In this study, we provide an overview of infant mortality trends in Shenyang, China from 1997 to 2014. During this period, infant mortality significantly decreased by 5.92% per year in this general region. The decreasing trends of overall infant mortality and categories of causes of infant death were more pronounced in urban areas than rural areas. Among categories of causes of infant death, congenital anomalies, asphyxia-related conditions, immaturity-related conditions, diseases of the nervous system and sense organs, and diseases of the respiratory system decreased significantly in the observational period. Additionally, the similar significant decreasing trends of urban and rural areas were observed among several causes of infant death.

Compared with the infant mortality rates in China (38 per 1,000 live births) that were provided by UNICEF^3^, the rates were significantly lower both in urban areas (8.49 per 1,000 live births) and rural areas (11.13 per 1,000 live births) of Shenyang. Although these rates were considerably lower than those of some Asian countries (e.g., India with 71 per 1,000 live births and Mongolia with 105 per 1,000 live births), they were higher than the infant mortality rates of Korea (6 per 1,000 live births) and Japan (4 per 1,000 live births)^3^. During the past two decades, according to the report of UNICEF that was published recently, the infant mortality rates of urban areas in 2013 were lower than that of the Republic of Korea (3 per 1,000 live births) and similar to that of Japan (2 per 1,000 live births)^3^. These substantial changes may be attributed to the efforts of the Chinese government. For example, with the ‘New Socialist Countryside’ policy in force, the Chinese government is establishing a rural cooperative medical and healthcare system, aimed at having over 80% of the rural population insured by 2010^5^. Additionally, the Chinese Ministry of Health has established a nationwide mortality surveillance network that collects children’s health information from representative population samples, to obtain accurate mortality estimates among Chinese newborns, infants, and children under the age of 5 years^5^. Although the Chinese government actively participated in the MDGs project [2], many of these efforts have been made to monitor and reduce the mortality of infants in the whole country, which results in the infant mortality rate continuously having decreased in the past decades. However, a report from the National Office for Maternal and Child Health Surveillance suggested that there were significant urban-rural differences of infant mortality rates in China^5^. Several studies presented that the infant mortality rates of coastal areas were obviously lower when compared with inland and remote areas^6^,^7^. For example, Zhang *et al.*^8^ have found that the infant mortality rate of Zhejiang province decreased from 11.99 per 1,000 live births to 3.89 per 1,000 live births from 1998 to 2011. Additionally, Huo *et al.*^7^ have reported that the infant mortality rate of Henan province decreased from 12.08 per 1,000 live births to 7.08 per 1,000 live births from 2004 to 2008. Herein, compared with our results, the infant mortality rate of these two provinces were relatively different^7^,^8^. Similarly, different magnitudes of decreasing trends were observed in the aforementioned areas. Further efforts should be taken to reduce the gap between the developed and the underdeveloped regions in China.

The first and second leading causes of infant mortality in Shenyang at the present time are congenital anomalies and immaturity-related conditions. Deaths due to these two categories decreased from approximately 3.41 to 0.56 per 1,000 live births and 2.30 to 1.22 per 1,000 live birth in the period between 1997 and 2014 in urban and rural areas, respectively (Table 1), which might be attributed to advances in obstetrics and neonatology in this period. The introduction and rapid dissemination of sonographic investigations during pregnancy and the subsequent changes in obstetric management of high-risk births are probably the main reasons for this improvement. Pregnancies with prenatal problems were earlier and more often detected, and thus the mothers could be transferred to specialized centers before giving birth^4^,^9^. Additionally, treatments for manageable disorders, such as congenital malformations of the heart, have been considerable improved, which increases patients’ survival rates^10^,^11^. Furthermore, because of the development difference between urban and rural areas, the APC of congenital anomalies and immaturity-related conditions of urban areas was smaller than that of rural areas (Table 3).

Among selective causes of infant mortality, the considerable decrease of the infant mortality caused by pneumonia during the observational period could be attributed to the substantial improvements of medical treatment in neonatal intensive care units and care during the perinatal period^12^. Except for preterm birth, low birth weight, and congenital heart disease, birth asphyxia was the major cause of infant mortality in urban and rural areas. Previous studies have suggested that decreasing infant mortality due to birth asphyxia mainly resulted from high rates of hospital delivery^5^. Compared with hospital delivery, home delivery, mainly assisted by a village doctor or midwife without sufficient knowledge and skills, is strongly linked with poverty, poor transport connection, or superstition^5^. In contrast to the 1.5% of neonatal deaths attributed to home delivery in urban areas, almost 25% of neonatal deaths in rural areas resulted from home delivery between 2003 and 2006. Therefore, during the past decades, the Chinese government has been implementing a subsidy project to allow rural pregnant women to give birth in hospitals, which reduces the infant mortality through increasing rural hospital delivery rates^13^.

The major strengths of the present study include the valuable evidence of the time trend of infant mortality from one of the most important developing countries. Compared with previous reports from other cities of China 6,7,8,14,15, the results of this study are comprehensive and representative: Shenyang is the provincial capital of Liaoning province, one of the largest provinces in China, and the highly reliable data from the Shenyang Women and Children Health Care Centre were used. Notably, we have carried out several analyses to quantify the time trends on the basis of a regression model, which method was rarely used in the previous other studies in China. Furthermore, the relatively longer observational period provides the possibility to describe the time trends of different causes of infant death. However, because there were limited or no cases of infant death due to several causes, we could not calculate the PC of these causes.

In summary, the findings in the present study are important for policy makers to understand the dynamics of infant mortality and potentially different decreasing trends between urban and rural areas in order to take appropriate actions to reduce this important public health burden.

## Material and Methods

### Study population

We analyzed 970,583 live births registered between 1997 and 2014 in the maternal and child health certificate registry managed by the Shenyang Women and Children Health Care Centre. This center is a comprehensive care institution and has been in charge of the women’s and children’s health care guidance in the city of Shenyang since 1987. This registry covers five urban districts (He Pin, Shen He, Da Dong, Huang Gu, and Tie Xi) and eight rural districts (Liao Zhong, Kang Pin, Fa Ku, Xin Ming, Shen Bei, Hun Nan, Yu Hong, and Su Jiatun), with approximately 8.1 million inhabitants. Infant mortality was defined as death of the infant between the date of birth and 365 days after birth, and we calculated the total number of deaths for all infants during the study period^16^.

### Data source

The main procedure for gathering data is passive notification of cases by public and private hospitals/clinics. Each case was evaluated by county health care institution-appointed pediatricians. The detailed procedures were described in the previous report^6^. Briefly, there have been two ways to collect the information through the Child and Maternal Surveillance System (CMSS). First, all hospitals in Shenyang must establish a death report card if infant dead in the hospital. The information of the death report card must be upload to the maternity and child care institution of each district. Subsequently, after checking these data, the maternity and child care institution of each district needs to upload data to the Shenyang Women and Children Health Care Centre. In order to guarantee the reporting rate as well as the quality of data, the Shenyang Women and Children Health Care Centre and the maternity and child care institution of each district will double check the original data in selective hospitals non-scheduled or every quarter. Secondly, for these infants did not die in the hospital, the community health service centers are require to establish the death report card and upload the data. Similarly, the Shenyang Women and Children Health Care Centre will double check the original data from these centers. Therefore, the reporting rate of infant deaths has been almost 100% so far. The data concerning the cause of infant deaths were classified according to the International Classification of Diseases (ICD)-10 codes. We obtained data regarding the infant deaths between January 1, 1997 and December 31, 2014. On the basis of previous reports^4^,^17^ and the International Collaborative Effort on Perinatal and Infant Mortality (ICE)^18^, we categorized the infant mortality into: congenital anomalies, asphyxia-related conditions, immaturity-related conditions, infections, diseases of the nervous system and sense organs, diseases of the respiratory system, diseases of the digestive system, and other causes. The unknown or missing causes of death were also officially classified into other causes of death. The rate of unknown or missing causes was less than 8% through the 17 years.

### Statistical analysis

Infant mortality rates were calculated for 17 1-year time intervals from 1997 to 2014. The annual percentage change for infant mortality rates was used to quantify the time trends^19^,^20^. A regression line was fitted to the natural logarithm of the rates, weighted by the number of cases, i.e. y = α + βx + ε, where y = ln (rate) and x = calendar year, and then the APC was calculated as 100 × (e^*β*^ − 1). The 95% confidence interval (CI) of the APC was calculated by the methods for population-based cancer statistics recommended by the National Cancer Institute^21^. All analyses were conducted using SPSS for Windows (version 17, SPSS Inc, Chicago, IL, USA). All statistical tests were two-sided, and *P*-values less than 0.05 were considered statistically significant.

## Additional Information

**How to cite this article**: Huang, Y.-H. *et al.* Different extent in decline of infant mortality by region and cause in Shenyang, China. *Sci. Rep.* **6**, 24527; doi: 10.1038/srep24527 (2016).

## Figures and Tables

**Table 1:** Infant mortality rates in urban and rural areas of Shenyang during 1997–2014.

Year	Urban and rural areas			Urban areas			Rural areas		
	Livebirths	No.	Rate*	Livebirths	No.	Rate*	Livebirths	No.	Rate*
1997	48628	462	9.50	30022	255	8.49	18606	207	11.13
1998	45881	425	9.26	27943	235	8.41	17938	190	10.59
1999	42474	374	8.81	26416	208	7.87	16058	166	10.34
2000	44245	409	9.24	28723	235	8.18	15522	114	7.34
2001	43244	397	9.18	28306	223	7.88	14938	174	11.65
2002	46163	424	9.18	30367	214	7.05	15796	210	13.29
2003	36015	356	9.88	15400	99	6.43	20615	257	12.47
2004	46240	360	7.79	28473	200	7.02	17767	160	9.01
2005	52972	448	8.46	22674	144	6.35	30298	304	10.03
2006	52237	420	8.04	22896	119	5.20	29341	301	10.26
2007	61111	471	7.71	30251	164	5.42	30860	307	9.95
2008	59163	350	5.92	30255	130	4.30	28908	220	7.61
2009	59232	281	4.74	32488	106	3.26	26744	175	6.54
2010	57523	283	4.92	34823	117	3.36	22700	166	7.31
2011	58303	268	4.60	37919	119	3.14	20384	149	7.31
2012	69469	259	3.73	49121	132	2.69	20348	127	6.24
2013	67856	272	4.01	49533	145	2.93	18323	127	6.93
2014	79827	251	3.14	61002	146	2.39	18825	105	5.58
Total	970583	6510	6.71	586612	2991	5.10	383971	3459	9.01

^*^Infant mortality rates were expressed as per 1000 live births.

**Table 2:** Trends in infant mortality rate of Shenyang during 1997–2014.

Cause of death	1997		2014		PC† (%)	APC† (%)	95% CI
	Case	Rate*	Case	Rate*			
Overall	462	9.50	251	3.14	−66.90	−5.92	−7.30, −4.51
Congenital anomalies	166	3.41	45	0.56	−83.49	−7.87	−9.81, −5.90
Asphyxia-related conditions	101	2.08	30	0.38	−81.91	−9.43	−10.19, −8.65
Immaturity-related conditions	112	2.30	97	1.22	−47.24	−3.44	−5.06, −1.79
Infections	3	0.06	13	0.16	163.97	−1.29	−6.58, 4.30
Nervous system and sense organs	9	0.19	6	0.08	−59.39	−6.01	−10.29, −1.52
Respiratory system	43	0.88	28	0.35	−60.33	−6.29	−9.03, −3.47
Digestive system	2	0.04	12	0.15	265.50	2.02	−3.45, 7.80

APC, annual percent change; CI, confidence interval; N/A, not available; PC, percent change.

^*^Infant mortality rates were expressed as per 1000 live births.

^†^Percent change and annual percent change between 1997 and 2014 was calculated by the infant mortality rate.

**Table 3:** Trends in infant mortality rate of Shenyang by urban and rural areas during 1997–2014.

Cause of death	Urban areas							Rural areas						
	1997		2014		PC† (%)	APC† (%)	95% CI	1997		2014		PC† (%)	APC† (%)	95% CI
	Case	Rate*	Case	Rate*				Case	Rate*	Case	Rate*			
Overall	255	8.49	146	2.39	−71.82	−7.41	−8.39, −6.42	207	11.13	105	5.58	−49.87	−3.92	−5.34, −2.48
Congenital anomalies	110	3.66	20	0.33	−91.05	−10.77	−12.83, −8.67	56	3.01	25	1.33	−55.88	−4.21	−6.22, −2.16
Asphyxia-related conditions	52	1.73	19	0.31	−82.02	−9.52	−11.04, −7.97	49	2.63	11	0.58	−77.81	−9.15	−10.68, −7.60
Immaturity-related conditions	51	1.70	58	0.95	−44.03	−4.11	−6.12, −2.06	61	3.28	39	2.07	−36.81	−2.37	−4.22, −0.49
Infectious	1	0.03	10	0.16	392.15	−2.96	−9.90, 4.52	2	0.11	3	0.16	48.25	0.50	−4.28, 5.52
Nervous system and sense organs	3	0.10	2	0.03	−67.19	−10.15	−14.97, −5.06	6	0.32	4	0.21	−34.11	−2.18	−7.23, 3.15
Respiratory system	22	0.73	18	0.30	−59.73	−7.32	−10.60, −3.92	21	1.13	10	0.53	−52.93	−4.78	−7.57, −1.91
Digestive system	1	0.03	8	0.13	293.72	1.92	−3.55, 7.69	1	0.05	4	0.21	295.35	1.11	−5.32, 7.97

APC, annual percent change; CI, confidence interval; N/A, not available; PC, percent change.

^*^Infant mortality rates were expressed as per 1000 live births.

^†^Percent change and annual percent change between 1997 and 2014 was calculated by the infant mortality rate.

**Table 4:** Trends in infant mortality rate of selective causes of Shenyang during 1997–2014.

Selective causes of death	1997		2014		PC† (%)	APC† (%)	95% CI
	Case	Rate*	Case	Rate*			
Pneumonia	41	0.84	24	0.30	−64.34	−5.45	−8.21, −2.60
Congenital heart disease	71	1.46	32	0.40	−72.54	−5.45	−7.43, −3.42
Neural tube defects	22	0.45	2	0.03	−94.46	−16.47	−19.94, −12.86
Preterm birth and low birth weight	77	1.58	73	0.91	−42.25	−2.18	−4.02, −0.29
Birth asphyxia	89	1.83	20	0.25	−86.31	−10.95	−11.89, −10.00
Intracranial hemorrhage of the newborn	28	0.58	4	0.05	−91.30	−10.33	−12.58, −8.02

APC, annual percent change; CI, confidence interval; PC, percent change.

^*^Infant mortality rates were expressed as per 1000 live births.

^†^Percent change and annual percent change between 1997 and 2014 was calculated by the infant mortality rate.

**Table 5:** Trends in infant mortality rate of selective causes of Shenyang by urban and rural area during 1997–2014.

Selective cause of death	Urban areas							Rural areas						
	1997		2014		PC† (%)	APC† (%)	95% CI	1997		2014		PC† (%)	APC† (%)	95% CI
	Case	Rate*	Case	Rate*				Case	Rate*	Case	Rate*			
Pneumonia	20	0.67	18	0.30	−55.71	−5.73	−9.07, −2.27	21	1.13	6	0.32	−71.76	−4.50	−7.29, −1.62
Congenital heart disease	43	1.43	14	0.23	−83.98	−8.61	−11.28, −5.85	28	1.50	18	0.96	−36.46	−1.00	−3.28, 1.34
Neural tube defects	14	0.47	0	0.00	−100.00	−15.04	−19.77, −10.04	8	0.43	2	0.11	−75.29	−15.04	−20.28, −9.46
Preterm birth and low birth weight	31	1.03	46	0.75	−26.97	−2.18	−4.43, 0.13	46	2.47	27	1.43	−41.99	−1.59	−3.65, 0.52
Birth asphyxia	47	1.57	11	0.18	−88.48	−11.22	−12.90, −9.51	42	2.26	9	0.48	−78.82	−10.42	−12.48, −8.30
Intracranial hemorrhage of the newborn	14	0.47	2	0.03	−92.97	−10.68	−14.74. −6.81	14	0.75	2	0.11	−85.88	−10.15	−13.33, −6.85

PC, percent change; APC, annual percent change; CI, confidence interval.

^*^Infant mortality rates were expressed as per 1000 live births.

^†^Percent change and annual percent change between 1997 and 2014 was calculated by the infant mortality rate.
